# Identification and characterization of the heme-binding proteins SeShp and SeHtsA of *Streptococcus equi *subspecies *equi*

**DOI:** 10.1186/1471-2180-6-82

**Published:** 2006-09-28

**Authors:** Tyler K Nygaard, Mengyao Liu, Michael J McClure, Benfang Lei

**Affiliations:** 1 Department of Veterinary Molecular Biology, Montana State University, Bozeman, Montana 59717, USA

## Abstract

**Background:**

Heme is a preferred iron source of bacterial pathogens. *Streptococcus equi *subspecies *equi *is a bacterial pathogen that causes strangles in horses. Whether *S. equi *has a heme acquisition transporter is unknown.

**Results:**

An *S. equi *genome database was blasted with the heme binding proteins Shp and HtsA of *Streptococcus pyogenes*, and found that *S. equi *has the homologue of Shp (designated SeShp) and HtsA (designated SeHtsA). Tag-free recombinant SeShp and SeHtsA and 6xHis-tagged SeHtsA (SeHtsA^His^) were prepared and characterized. Purified holoSeShp and holoSeHtsA bind Fe(II)-protoporphyrin IX (heme) and Fe(III)-protoporphyrin IX (hemin) in a 1:1 stoichiometry, respectively, and are designated hemoSeShp and hemiSeHtsA. HemiSeShp and hemiSeHtsA^His ^can be reconstituted from apoSeShp and apoSeHtsA^His ^and hemin. HemoSeShp is stable in air and can be oxidized to hemiSeShp by ferricyanide. HemiSeHtsA can be reduced into hemoSeHtsA, which autoxidizes readily. HemoSeShp rapidly transfers its heme to apoSeHtsA^His^. In addition, hemoSeShp can also transfer its heme to apoHtsA, and hemoShp is able to donate heme to apoSeHtsA^His^.

**Conclusion:**

The primary structures, optical properties, oxidative stability, and in vitro heme transfer reaction of SeShp and SeHtsA are very similar to those of *S. pyogenes *Shp and HtsA. The data suggest that the putative cell surface protein SeShp and lipoprotein SeHtsA are part of the machinery to acquire heme in *S. equi*. The results also imply that the structure, function, and functional mechanism of the heme acquisition machinery are conserved in *S. equi *and *S. pyogenes*.

## Background

Iron is an essential nutrient that facilitates a wide variety of vital biological functions in almost all organisms. The ability of almost all pathogenic bacteria to acquire iron from the host environment is essential to establish infection [1]. Under the physiological conditions the predominant species of free iron, Fe^3+^, has a very low solubility and is of insufficient quantity to sustain bacterial growth. During infection, the mammalian host further deprives invading microorganisms of available iron through a process known as the hypoferraemic response [2]. Consequently, pathogenic bacteria must acquire iron from alternate sources and/or overcome the low solubility of Fe^3+ ^by producing siderophores [3], whose complexes with Fe^3+ ^is taken up by specific transporters.

Heme bound to hemoproteins constitutes the major iron reservoir in mammalian hosts and is the preferred iron source of bacterial pathogens [4]. Heme may be an even more important iron source for some streptococci. Unlike many other pathogenic bacteria, no streptococci have been reported to produce siderophores [5], though it was recently shown that the ABC transporter FtsABCD of *Streptococcus pyogenes *takes up ferric ferrichrome [6]. Free heme and hemoproteins, but not Fe-loaded transferrin nor lactoferrin, can be used as the iron source of *S. pyogenes *[7].

Different mechanisms for heme acquisition have been demonstrated in bacteria. Gram-negative bacteria *Serratia marcescens*, *Pseudomonas aeruginosa*, *Pseudomonas fluorescens*, *Haemophilus influenza*, and *Yersinia pestis *secrete proteins called hemophores to extract heme from host hemoproteins and deliver it to the receptor in the outer membrane [8]. The caught heme is then transported across the outer membrane into the periplasm in a TonB-dependent process [9] and finally brought across the cytoplasmic membrane by heme-specific ATP-binding cassette (ABC)-containing transporter. Some Gram-negative bacteria also acquire heme in a hemophore-independent mechanism. Specific outer membrane receptor directly interacts with and extracts heme from hemoproteins. Hemophore has not been found in Gram-positive bacteria. In addition to heme specific ABC transporters [10-12], cell surface proteins binding hemoproteins and heme have been identified in Gram-positive pathogens [13,14]. It is believed that these surface proteins scavenge and relay heme from host hemoproteins to ABC transporter [15,16].

The machinery involved in heme acquisition in Gram-positive bacteria is not conserved. *S. pyogenes *produces a cell surface heme-binding protein Shp [13]. This protein does not show any significant homology to the cell surface heme-binding proteins of *Staphylococcus aureus *[14]. The *shp *and *htsABC *genes, which encode heme-specific ABC transporter, are co-transcribed with the 6 other genes and regulated by MtsR in response to iron levels [11,17,18]. We recently demonstrated that Shp rapidly transfers its heme to apoHtsA by a direct and affinity-driven mechanism [15,16].

*Streptococcus equi *subspecies *equi*, a member of Gram-positive group C streptococci, causes strangles in horses, one of the most common infectious diseases of equine [19]. A metal binding protein (MBL) has been detected in *S. equi *[20], which is also a homologue of MtsA of *Streptococcus pyogenes *manganese and iron transporter MtsABC [21]. However, very little is known about iron acquisition in *S. equi*. *S. equi *has the homologue of Shp and HtsABC (designated SeShp and SeHtsABC, respectively. For simplicity, Shp and HtsA refer to the *S. pyogenes *proteins in this report.). As an initial effort to understand the iron acquisition processes in *S. equi*, we prepared and characterized SeShp and SeHtsA. The results suggest that the machinery and mechanism of heme acquisition are conserved in *S. equi *and *S. pyogenes*.

## Results

### S. equi shp and htsA genes

**Figure 1:**
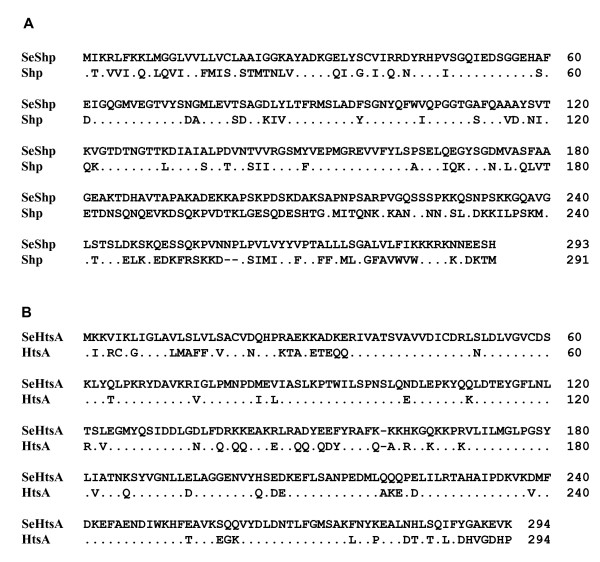
**Amino acid sequence alignments of *S. pyogenes *Shp and *S. equi *SeShp (A) and SeHtsA and HtsA (B)**. Amino acid residues of each *S. pyogenes *protein identical to those of the corresponding *S. equi *protein (.) and gaps introduced to maximize alignment (-) are indicated.

A blast search of available *S. equi *genome [22] was conducted to find out whether *S. equi *has a heme acquisition system homologous to the one in *S. pyogenes*. *S. equi *homologues of *shp *and *htsABC *are present. SeShp and Shp encoded by *spy1796 *are highly conserved in the N-terminal half of the mature form, sharing 75% identity and 91% similarity in amino acid sequence in the region of amino acid residues 28 through 173 amino acids. In contrast, the secretion signal sequences and the C-terminal half share less than 25% identity (Fig. 1A), suggesting that the N-terminal half of SeShp and Shp is important to their function. Unlike SeShp, SeHtsA shares with HtsA, which is encoded by *spy1795*, 76% identity in amino acid sequence throughout the whole proteins (Fig. 1B). The 5 downstream genes cotranscribed with *shp *and *htsABC *[13] also have their homologues next to *S. equi shp *and *htsABC*, indicating that the locus is conserved in *S. pyogenes *and *S. equi*.

### Recombinant SeShp and SeHtsA proteins

**Figure 2:**
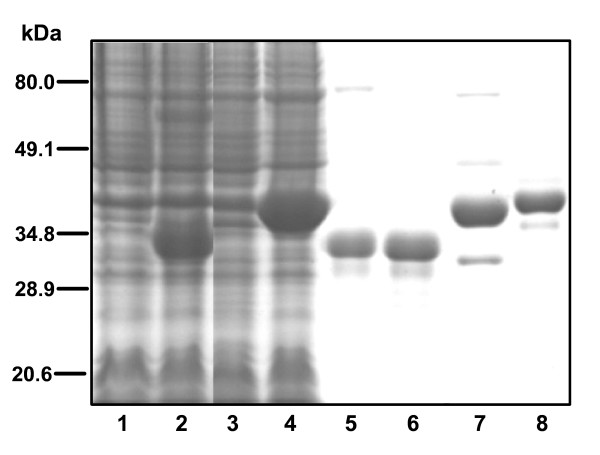
**SDS-PAGE analysis of recombinant holoSeShp, apoSeShp, SeHtsA, and SeHts^His^**. SeShp lacks its secretion signal sequence and C-terminal transmembrane domain and charged tail, and SeHtsA and SeHts^His ^lacks the secretion signal sequence and lipid moiety. The gel was stained with GelCode blue. Samples shown are *E. coli *with vector (control) (lane 1), *E. coli *lysate containing SeShp (2); SeHtsA (3), and SeHtsA^His ^(4), and purified holoSeShp (5), apoSeShp (6), SeHtsA (7), and SeHtsA^His ^(8).

The existence of the *shp *and *htsABC *homologues in *S. equi *suggests that the pathogen may have a heme acquisition system similar to the one in *S. pyogenes*. If this is true, SeShp and SeHtsA should bind heme. To test this idea, recombinant mature SeShp, without the C-terminal transmembrane domain and charged tail, and SeHtsA, without lipid moiety, were prepared. Tag-free SeShp was overexpressed in *E. coli*, while tag-free SeHtsA was produced at a much lower level (Fig. 2). Fusion of the 6xHis tag to SeHtsA (SeHtsA^His^) at the amino terminus greatly enhanced yield of SeHtsA. SeShp, SeHtsA, and SeHtsA^His ^were purified to more than 90% purity based on SDS-PAGE (Fig. 2).

**Figure 3:**
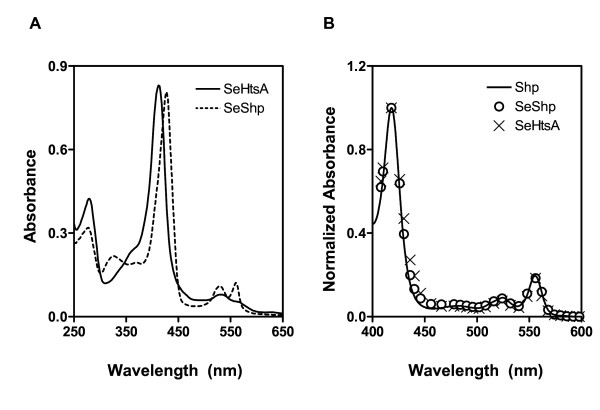
**SeShp and SeHtsA bind heme**. (A) UV-visible absorption spectra of 9 μM SeShp (dashed line) and SeHtsA (solid line) in 20 mM Tris-HCl, pH 8.0. (B) Spectra of reduced pyridine hemochrome derived from Shp (positive control) (solid line), SeShp (open circles), and SeHtsA (crosses). The spectra were normalized for comparison.

Expression of SeShp, SeHtsA, or SeHtsA^His ^turns *E. coli *pellet red, the same phenomenon observed in the production of Shp and HtsA in *E. coli*, suggesting that SeShp and SeHtsA also bind heme (Fe(II)-protoporphyrin IX). Purification of SeShp results in two forms, one with red color and another without color. The red SeShp fraction has a spectrum with absorption peaks at 428, 530 and 560 nm in the visible region (Fig. 3A). This spectrum is identical to that of heme-containing Shp, suggesting that SeShp binds heme. A pyridine hemochrome assay was used to further determine whether the chromophore associated with SeShp is heme. The spectrum of SeShp in the assay is identical to that of the pyridine hemochrome derived from Shp, displaying peaks at 418, 524, and 556 nm (Fig. 3B), confirming that SeShp binds heme. Measurements of heme and protein contents found that each SeShp binds 0.9 heme in the red SeShp sample. These results indicate that SeShp binds heme in a 1:1 stoichiometry.

**Figure 4:**
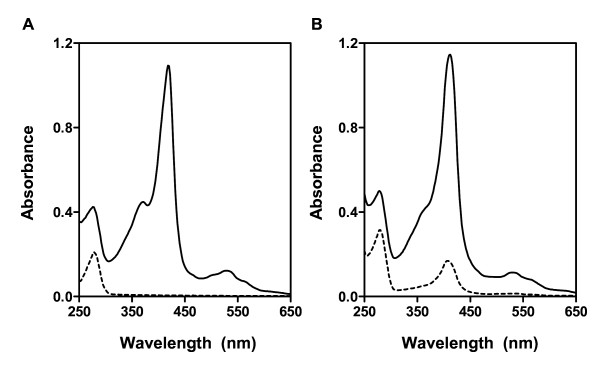
**Reconstitution of holoSeShp (A) and holoSeHtsA^His ^(B) by hemin**. Purified apoSeShp and 82% apoSeHts^His ^were incubated with hemin and separated from free hemin by gel filtration using a Sephadex G-25 column. Displayed are the optical absorption spectra of the untreated (dashed curves) and treated (solid curves) protein samples. Concentrations of SeShp and SeHtsA^His ^were 10 μM and 13 μM, respectively.

Both SeHtsA and SeHtsA^His ^purified are red, and the spectrum of SeHtsA shows an absorption peak at 410 nm (Fig. 3A), which is similar to the spectrum of hemiHtsA. HemiHtsA binds the hexacoordinate hemin (Fe(III)-protoporphyrin IX), suggesting that SeHtsA also bind hemin. The spectrum of reduced pyridine hemochrome derived from SeHtsA was identical to that derived from Shp, confirming that SeHtsA indeed binds hemin (Fig. 3B). Measurements of hemin and protein contents found that purified SeHtsA binds 0.97 heme per SeHtsA molecule, indicating a 1:1 stoichiometry for hemin binding. SeHtsA displays a much higher ratio of the Soret absorption peak over the absorbance at 280 nm (Fig. 3A) than SeHtsA^His ^(Fig. 4B) at a same protein concentration, indicating that not all SeHts^His ^had bound hemin. Hemin- and protein-content measurements found that the molar ratio of bound hemin over SeHtsA^His ^protein was 0.18. These results indicate that 82% of SeHtsA^His ^was in a heme-free or apo-protein form.

### Reconstituted SeShp- and SeHtsA^His^-hemin complexes

If SeShp and SeHtsA bind heme and hemin, holoSeShp and holoSeHtsA should be reconstituted from apo-proteins and hemin. ApoSeShp and SeHtsA^His ^containing 82% apo-form were incubated with hemin, and passed through a G-25 column to separate proteins from free hemin. ApoSeShp does not have absorption in the visible region, while the treated SeShp has an absorption peak at 418 nm, the Soret absorption peak of bound hemin (Fig. 4A). Measurements of protein and hemin contents found that the treated SeShp bound 1.08 hemin per protein molecule. The absorbance of reconstituted SeHtsA^His ^at 408 nm was 4.2 times as that of untreated SeHtsA^His ^at the same protein concentration (Fig. 4B). As assessed by the pyridine hemochrome and protein concentration assays, reconstituted SeHtsA^His ^bound 1.01 hemin per protein molecule. These results indicate that hemiSeShp and hemiSeHtsA^His ^are reconstituted from hemin and apo-proteins, demonstrating that both proteins bind hemin in a 1:1 stoichiometry.

### Oxidation states of protoporphyrin Fe in SeShp and SeHtsA

**Figure 5:**
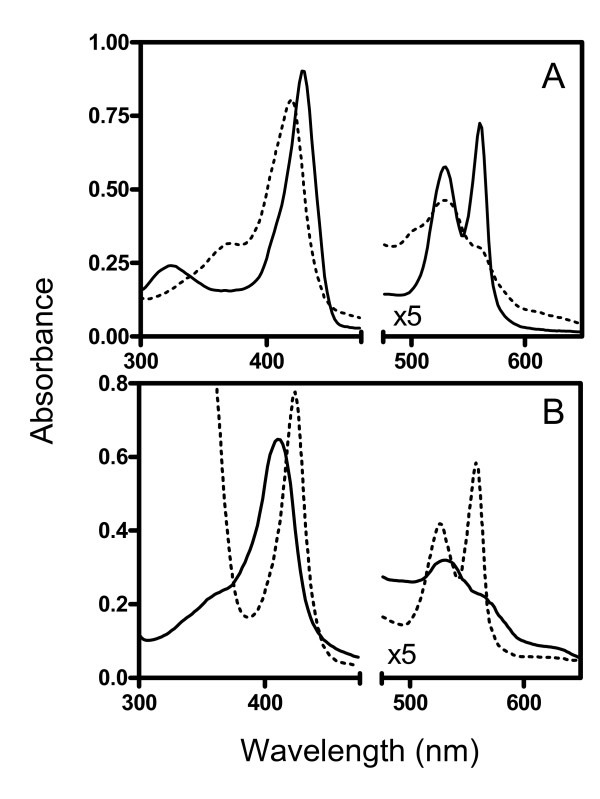
**Conversion between and stability of oxidation states of SeShp and SeHtsA^His^**. (A) Optical absorption spectra of 6 μM hemiSeShp from the oxidation of hemoSeShp with ferricyanide (dashed curve) and hemoSeShp obtained from the reduction of hemiSeShp with dithionite (solid curve). The hemoSeShp spectrum was recorded after the removal of excess dithionite. (B) Optical absorption spectra of 5 μM dithionite-treated holoSeHtsA^His ^in the presence of excess dithionite (dashed line) and after the removal of dithionite (solid line).

The spectrum of purified holoSeShp has a dominant α peak at 560 nm and resembles the spectra of reduced b-type cytochrome with a 6-coordinate heme iron and hemochrome, indicating that purified holoSeShp is in the hexacoordinate heme or hemochrome form (designated hemoSeShp). In contrast, holoSeShp reconstituted from apoSeShp and hemin shows a typical spectrum of hemichrome or oxidized b-type cytochrome with a 6-coordiate hemin iron, implying that the reconstituted holoSeShp is in the hemichrome form (designated hemiSeShp). These results indicate that purified holoSeShp is in the reduced state, which is resistant to autoxidation. The following results further confirm this conclusion. The treatment of hemoSeShp with ferricyanide results in the hemichrome visible spectrum, and hemoSeShp is formed upon the addition of dithionite to hemiSeShp and remains in the hemochrome form after the removal of excess dithionite (Fig. 5A). The results demonstrate that the ferric to ferrous and ferrous to ferric transition can be readily achieved by the treatments with the reducing and oxidizing agents, respectively.

Both purified SeHtsA and reconstituted SeHtsA^His ^are in the hemichrome form. The addition of excess dithionite to hemiSeHts^His ^instantly leads to the formation of hemoSeHtsA^His^, as evidenced by the shift of the Soret peak from 410 to 424 nm and the replacement of the absorption peak of hemichrome at 530 nm with the hemochrome specific peaks at 526 and 558 nm (Fig. 5B). However, the removal of excess dithionite by gel filtration results in only hemiSeHtsA^His^, indicating that hemoSeHtsA is oxidized by O_2_.

### Heme transfer from hemoSeShp to apoSeHtsA^His^

**Figure 6:**
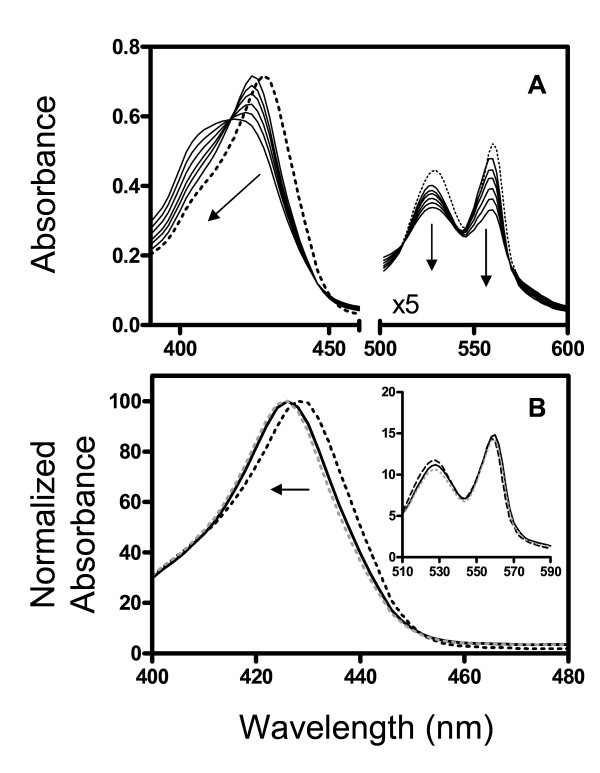
**Spectral evidence for heme transfer from hemoSeShp to apoSeHtsA^His^**. (A) The shift over time in the visible spectrum of a mixture containing 4 μM hemoSeShp and 6 μM SeHtsA^His^. The spectra (solid curves) were taken at 0, 90, 165, 315, 540, 990 seconds after mixing. The dashed line is the sum of the spectra of 4 μM hemoSeShp and 6 μM SeHtsA^His^. (B) The spectral change in the visible region after mixing 4 μM hemoSeShp and 6 μM SeHtsA^His ^with excess dithionite. The spectra were recorded immediately (solid curve) and 6 min after mixing (dotted grey curve). The spectrum of hemoSeShp is included as a reference for change (dashed curve). The spectra were normalized for comparison. Insert: the same spectra in the region from 510 to 590 nm. The arrows indicate the directions of the spectral changes with time.

The spectral changes are associated with the heme transfer from Shp to apoHtsA and the subsequent autoxidation of the resulting hemoHtsA [15,16]. The changes have been used to examine the mechanism of the heme transfer. To determine whether hemoSeShp also transfer its heme to apoSeHtsA^His^, the spectrum of a hemoSeShp/apoSeHtsA mixture was recorded with time after mixing. Upon mixing, the Soret absorption peak instantly shifts from 428 to 424 nm. Following the rapid initial shift, a slower spectral change occurs during which the Soret absorption peak gradually shifts towards 410 nm, and, at the same time, the 524 nm and 556 nm peaks decrease (Fig. 6A). Similar rapid and slow spectral changes are observed after mixing hemoShp and apoHtsA and are due to the formation and subsequent autoxidation of hemoHtsA [16]. If the slower change in Fig. 6A is due to the autoxidation of hemoSeHtsA^His^, mixing the proteins under anaerobic conditions should lack the slow spectral change. These were what happened when hemoSeShp and apoSeHtsA^His ^were mixed in the presence of excess sodium dithionite (Fig. 6B). The data indicate that the heme transfer from hemoSeShp to apoSeHtsA^His ^follows the same mechanism of the heme transfer from hemoShp to apoHtsA.

### Heme transfer from hemoSeShp to apoHtsA and from hemoShp to apoSeHtsA

**Figure 7:**
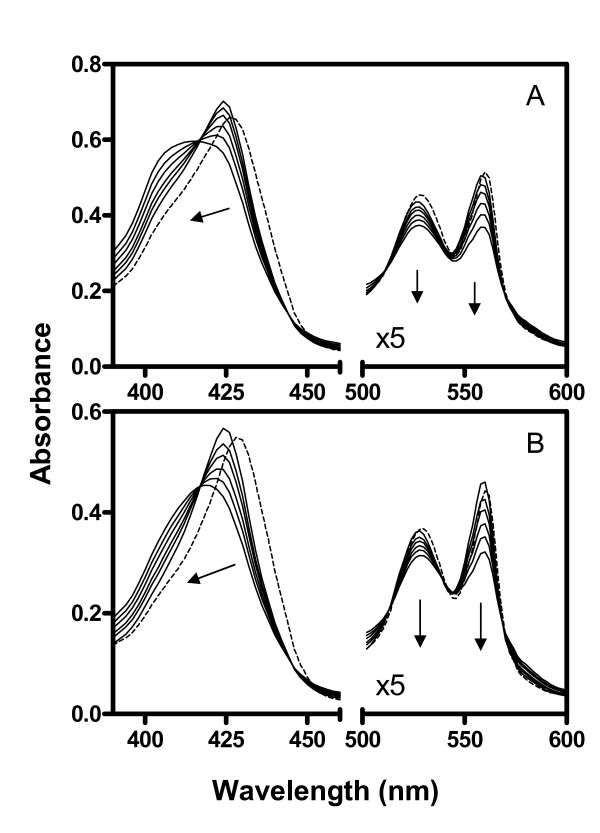
**Heme transfer from hemoSeShp to apoHtsA and from hemoShp to apoSeHtsA^His^**. Presented are the shift in the visible spectra of the mixtures of 4 μM hemoShp/6 μM apoSeHtsA^His ^(A) and 4 μM hemoSeShp/6 μM apoHtsA (B) immediately and 90, 165, 315, 540, and 990 seconds after mixing. The dashed line in each panel is the sum of the spectra of the two individual proteins at the same concentrations. The arrows indicate the directions of the spectral changes with time.

If the structures, functions, and functional mechanism of the heme acquisition machinery are well conserved in *S. equi *and *S. pyogenes*, hemoSeShp and hemoShp should be able to transfer their heme to apoHtsA and apoSeHtsA, respectively. To test this idea, the heme transfer experiment was performed for the combinations of hemoSeShp/apoHtsA and hemoShp/apoSeHtsA. In both cases, the rapid shift in the Soret absorption peak and the subsequent slow change are observed (Fig. 7), indicating that hemoSeShp and hemoShp each can transfer heme to both apoSeHtsA and apoHtsA.

## Discussion

In this study, we have identified the putative cell surface protein SeShp and lipoprotein SeHtsA as heme binding proteins. We have demonstrated that hemoSeShp can rapidly transfer its heme to apoSeHtsA. The results suggest that the structures, functions, and functional mechanisms of the heme acquisition machinery in *S. equi *and *S. pyogenes *are conserved.

Three evidences support the conclusion that SeShp and SeHtsA bind heme and hemin. First, the purified holoSeShp and holoSeHtsA possess the absorption spectra similar to those of the reduced and oxidized b-type cytochrome or the hemochrome and hemichrome, respectively [23,24]. Secondly, pyridine hemochrome analysis of SeShp and SeHtsA results in reduced pyridine hemochrome. Thirdly, the hemichrome complexes are formed from the reaction of apoSeShp or apoSeHtsA^His ^and hemin. It is interesting that the purified holoSeShp shows the dominant α absorbance band at 560 nm, which is only seen for reduced b-type cytochrome and hemochrome. The treatment of hemoSeShp with ferricyanide converts it into hemiSeShp, and reduction of hemiSeShp by dithionite turns it back to hemoSeShp. Thus, hemoSeShp is unusually stable in air, though the basis for the stability is unknown. In contrast to hemoSeShp, hemoSeHtsA is readily oxidized by oxygen.

The initial rapid and subsequent slower spectral changes upon mixing hemoShp and apoHtsA have been shown to be associated with the formation of hemoHtsA and its autoxidation, respectively [16]. The spectral changes observed after mixing hemoSeShp and apoSeHtsA are very similar to those that occurred when mixing hemoShp and apoHtsA, indicating that hemoSeShp also rapidly transfers its heme to apoSeHtsA and the resulting hemoSeHtsA autoxidizes. This interpretation is supported by the fact that the slower spectral change in the reaction of hemoSeShp and apoSeHtsA^His ^is absent when dithionite is present.

Our data suggest that *S. equi *and *S. pyogenes *use similar machinery to acquire heme. SeShp and Shp or SeHtsA and HtsA share high homology in amino acid sequence. These homologous proteins have similar absorption spectra associated with bound heme and hemin and similar stability in oxidation states in air. More importantly, like Shp, SeShp can rapidly transfer its heme to apoHtsA. These data suggest that the heme acquisition machinery and mechanism in both pathogens are conserved. This assertion is further supported by the fact that heme can be transferred from hemoSeShp to apoHtsA and from hemoShp to apoSeHtsA.

The high homology between SeShp and Shp is located in the N-terminal half of the proteins. We propose that the N-terminal half is involved in heme binding and transfer while the C-terminal half functions as a spacer to place the N-terminal part beyond the cell wall. Blast of GenBank does not reveal other proteins homologous to SeShp and Shp, suggesting that SeShp and Shp contain a novel heme-binding pocket, which renders the stability of hemochrome in air. In contrast, SeHtsA also share homology with FatB encoded by the genomes of *Listeria monocytogenes, Staphylococcus aureus*, *Bacillus anthracis*, and *Clostridium perfringens*. FatB is annotated as a putative ferric ferrichrome binding protein of ABC transporters. The fact that SeHtsA and HtsA bind heme suggests that these SeHtsA homologues may be heme-binding proteins and part of the transporters for heme acquisition.

## Methods

### Materials and strain

Sephadex G-25, SP sepharose and DEAE sepharose were purchased from Amersham (Piscataway, NJ). Nickel-nitrilotriacetic acid agarose (Ni-NTA) was obtained from QIAGEN (Valencia, CA). Bovine hemin chloride was from Sigma (St. Louis, MO). *S. equi *strain SEM1 was isolated from a horse suffering strangles in Montana, USA in 2003.

### Sequence alignment

Blast search of the *Streptococcus equi *genome [25] and GenBank with the Shp and HtsA amino acid sequences were carried out to identify their homologues. Sequence alignment was performed using multiple sequence alignment by CLUSTALW under default parameters [26].

### Gene cloning

*S. equi shp *and *htsA *were cloned from strain *S. equi *strain SEM1 using primer pairs 5'-AccatggATAAGGGTGAGCTTTATTCC-3'/5'-CgaattcTAATTATTTACTGGCTTTTGGGAC-3' and 5'-AccatggGTGTGGATCAGCACCCTAG-3'/5'-CgaattcTTACTTGACTTCCTTTGCTCC-3', respectively. The ccatgg and gaattc in lower case in the primers are introduced NcoI and EcoRI sites, respectively, and the sequences underlined correspond to the respective genes. The PCR products were digested with *Nco*I and *Eco*RI and ligated into pET-21d at the *Nco*I and *Eco*RI sites to yield pSEHTSA and pSESHP for *S. equi htsA *and *shp *genes, respectively. SeHtsA produced from pSEHTSA is tag-free mature protein with its first residue Cys20 replaced with Gly. SeShp produced from pSESHP is tag-free protein without the secretion signal sequence (residues 1–29) and lacks the transmembrane domain and charged tail (amino acids 260 to 293) located at the carboxyl terminus. The *S. equi htsA *PCR product was also cloned into pET-His2 [11] at the *Nco*I and *Eco*RI sites to yield pSEHTSA2, which encodes SeHtsA^His ^with a 6xHis tag (MHHHHHHLETMG) fused to the second amino acid residue of mature SeHtsA. The cloned genes have the identical sequences with those in the *S. equi *genome database [22].

### Protein expression

SeShp, SeHtsA, and SeHtsA^His ^were expressed in *Escherichia coli *BL21(DE3) containing the corresponding plasmid. *E. coli *cells were grown at 37°C in 3 L Luria-Bertani broth supplemented with 100 mg of ampicillin/L. When the optical density at 600 nm reached approximately 1.0, the expression was induced with 0.5 mM isopropyl β-D-thiogalactopyranoside for 6 hours and harvested by centrifugation.

### Protein purification

All solutions were buffered with 20 mM Tris-HCl, pH 8.0 (Tris-HCl). To prepare samples for column chromatography, bacterial pellets containing expressed protein were resuspended in 40 ml Tris-HCl, sonicated on ice for 15 min, and centrifuged at 20,000 × *g *for 10 min to remove cell debris. All dialysis was performed against 3 l Tris-HCl at 4°C for 12 hours with one buffer change. Purified proteins were concentrated using Centricon Plus 20 filtration devices (Millipore, Bedford, MA). Shp and HtsA of *S. pyogenes *were prepared as previously described [11,13].

To purify holoSeShp, the lysate containing SeShp was loaded onto a DEAE column (2.5 × 6 cm), washed with 100 ml Tris-HCl, and bound SeShp was eluted with a 100-ml gradient of 0 – 0.3 M NaCl in Tris-HCl. Eluted fractions were analyzed using SDS-PAGE and those containing holoSeShp and apoSeShp were separately pooled and dialyzed. The holoSeShp pool was loaded onto an SP sepharose column (1.5 × 20 cm) and washed with 80 ml Tris-HCl, and the column was eluted with a 100-ml gradient of 0–0.15 M NaCl in Tris-HCl. Fractions containing holoSeShp with >90% of purity were pooled, dialyzed, concentrated, and stored at -20°C prior to use. To prepare apoSeShp, the apoSeShp pool was loaded onto a Q sepharose column (1.5 × 20 cm), and the column was eluted with a 0–0.3 M gradient of NaCl. The fractions containing SeShp were pooled, adjusted to 1.0 M (NH_4_)_2_SO_4_, and loaded onto a 1.5 × 5.0-cm phenyl sepharose column. The column was washed with 50 ml 1.0 M (NH_4_)_2_SO_4 _and eluted with an 80-ml linear gradient of 1.0 to 0.25 M gradient of (NH_4_)_2_SO_4_.

To prepare SeHtsA, the lysate containing SeHtsA was loaded onto a 2.5 × 10-cm column of DEAE sepharose. The column was washed with Tris-HCl and eluted with a 150-ml linear gradient of 0–0.1 M NaCl. Fractions containing relatively pure SeHtsA were pooled, adjusted to 1.0 M (NH_4_)_2_SO_4_, and loaded onto a 1.5 × 6-cm column of phenyl sepharose. The column was washed with 50 ml 1.0 M (NH_4_)_2_SO_4 _and eluted with a 40-ml linear gradient of 1.0–0.6 M (NH_4_)_2_SO_4_. Fractions containing SeHtsA were pooled, dialyzed, and subjected to the DEAE Sepharose chromatography again as described above. Fractions containing SeHtsA with >90% purity were pooled, dialyzed, and concentrated using a Centricon Plus 20 filter device.

To purify SeHtsA^His^, the lysate containing SeHtsA^His ^was loaded onto a Ni-NTA column (2.5 × 6 cm), and the column was washed with 100 ml Tris-HCl and eluted with 0.15 M imidazole in Tris-HCl. Fractions positive with SeHtsA^His^, as assessed by SDS-PAGE, were pooled and dialyzed. The dialyzed sample was loaded onto a DEAE sepharose column (2.5 × 5 cm), and the column was washed with 80 ml Tris-HCl and eluted with 0.10 M NaCl Tris-HCl. Fractions containing SeHtsA^His ^were pooled, dialyzed, concentrated, and stored at -20°C prior to use.

### Assays for protein and heme or hemin contents

Protein concentrations were determined using a modified Lowry protein assay kit (Pierce, Rockford, IL) with bovine serum albumin as a standard. Heme or hemin content was measured using a pyridine hemochrome assay as follows [27]. Protein sample was added in 750 μl Tris-HCl and mixed with 75 μl 1 N NaOH, 175 μl pyridine, and approximately 2 mg sodium dithionite. The optical absorption spectrum was immediately recorded. The heme or hemin content was determined using the extinction coefficient ε_418 _= 191.5 mM^-1 ^cm^-1^.

### Reconstitution of hemiSeShp and hemiSeHtsA^His^

HemiSeShp and hemiSeHtsA^His ^were reconstituted from apoSeShp and 82% apoSeHts^His^, respectively, with bovine hemin chloride as described previously [11]. Briefly, each protein was incubated at room temperature with hemin in 1.0 ml Tris-HCl at a protein:hemin ratio of 1:2 for 15 minutes. The sample was then loaded onto a Sephadex G-25 column (1.5 × 20 cm), and the holoprotein was eluted with Tris-HCl.

### Reduction and oxidation of holoSeShp and holoSeHtsA^His^

Reduction of hemiSeShp and hemiSeHtsA^His ^was achieved by adding approximately 1 mg sodium dithionite to 200 μl protein. In the specified cases, excess dithionite was removed from the proteins by passing the samples through a Sephadex G-25 column (1.5 × 20 cm). Oxidation of hemoSeShp was achieved by the reaction with ferricyanide. Ferricyanide (1 mM) was added to 200 μl hemoSeShp, and hemiSeShp formed was separated from excess ferricyanide using a Sephadex G-25 column (1.5 × 20 cm).

### Heme transfer

Transfer of heme from hemoSeShp or hemoShp to SeHtsA^His ^(82% apo form and 18% holo form) or apoHtsA was followed by monitoring the spectral changes, as described previously [16]. Briefly, the holo- and apo-proteins were mixed at the indicated concentrations in 100 μl Tris-HCl at 25°C, and the optical absorption spectra of the samples were recorded at indicated times. Heme transfer under reduced conditions was achieved by addition of excess sodium dithionite to both apo and holo proteins prior to mixing. All absorption spectra were obtained using a SPECTRAmax 384 Plus spectrophotometer (Molecular Devices, Sunnyvale, CA).

### Nucleotide sequence accession numbers

The GenBank accession numbers for the nucleotide sequences of the *Streptococcus equi shp *and *htsA *genes are DQ975461 and DQ975462, respectively.

## Authors' contributions

TKN purified hemoSeShp and SeHtsA^His^, characterized the proteins, and drafted part of the manuscript. ML cloned the genes. MJM purified SeHtsA. BL designed the study and prepared the manuscript. All authors have read and approved the final manuscript.
